# Effect of Neonicotinoids on Bacterial Symbionts and Insecticide-Resistant Gene in Whitefly, *Bemisia tabaci*

**DOI:** 10.3390/insects12080742

**Published:** 2021-08-18

**Authors:** Mritunjoy Barman, Snigdha Samanta, Himanshu Thakur, Swati Chakraborty, Arunava Samanta, Amalendu Ghosh, Jayanta Tarafdar

**Affiliations:** 1 Department of Agricultural Entomology, BCKV, Mohanpur 721436, India; barman.mritunjoy@bckv.edu.in (M.B.); snigdhamanu23@gmail.com (S.S.); asamanta64@yahoo.co.in (A.S.); 2 Department of Entomology, C.S.K. Himachal Pradesh Krishi Vishvavidyalaya, Palampur 176062, India; himanshumaimor@gmail.com; 3 Department of Plant Pathology, BCKV, Nadia, Kalyani 741245, India; swatichak777.sc@gmail.com; 4 Division of Plant Pathology, ICAR-Indian Agricultural Research Institute, New Delhi 110012, India; amal4ento@gmail.com; 5 Directorate of Research, BCKV, Kalyani 741235, India

**Keywords:** qPCR, gene expression, cytochrome P450, *Portiera*, *Rickettsia*, management

## Abstract

**Simple Summary:**

The silverleaf whitefly (*B. tabaci*) is an important agricultural pest damaging several agricultural and horticultural crops worldwide. Keeping in mind the status of insecticide overuse, the current experiment was designed to evaluate the sensitivity of *B. tabaci* towards imidacloprid and thiamethoxam (two popularly used neonicotinoids in India). The lab population of *B. tabaci* was found to be more susceptible to thiamethoxam compared to imidacloprid. qPCR studies revealed a higher expression of insecticide-resistant genes, *CYP6CM1* and *CYP6CX1*, after imidacloprid treatment that might be responsible for increased resistance to insecticides. Our results also put forward the different interactions between symbionts and insecticide resistance. qPCR studies revealed thiamethoxam-treated *B. tabaci* populations harbored a higher amount of primary endosymbiont *Portiera* and a lower amount of secondary symbiont *Rickettsia*.

**Abstract:**

The silverleaf whitefly, *Bemisia tabaci* (Gennadius, Hemiptera: Aleyrodidae), is a major threat to field and horticultural crops worldwide. Persistent use of insecticides for the management of this pest is a lingering problem. In the present study, the status of sensitivity of *B. tabaci* to two neonicotinoids, imidacloprid and thiamethoxam, was evaluated. The expression pattern of two cytochrome P450 (*cyp*) genes and changes in the relative amount of symbionts in insecticide-treated *B. tabaci* were also assessed. Quantitative PCR (qPCR) studies indicate that the *CYP6CM1* and *CYP6CX1* genes were always expressed higher in imidacloprid-treated whitefly, suggesting a correlation between gene expression and the insect’s ability to detoxify toxic compounds such as insecticides. In addition, the thiamethoxam-treated population harbored higher *Portiera* and lower *Rickettsia* titers, whereas the imidacloprid-treated population harbored more *Rickettsia* at different time intervals. Interestingly, we also examined that an increase in exposure to both the insecticides resulted in a reduction in the mutualistic partners from their insect host. These differential responses of endosymbionts to insecticide exposure imply the complex interactions among the symbionts inside the host insect. The results also provide a deeper understanding of the molecular mechanism of resistance development that might be useful for formulating effective management strategies to control *B. tabaci* by manipulating symbionts and detoxifying genes.

## 1. Introduction

The whitefly *Bemisia tabaci* (Gennadius, Hemiptera: Aleyrodidae) is a polyphagous pest that inflicts damage by direct feeding on phloem sap or by vectoring a large number of plant viruses [1,2]. It is a major threat to various field and horticultural crops worldwide. *B. tabaci* consists of a complex of biotypes, which vary largely with respect to characters such as host range, insecticide resistance, fecundity, and their ability to transmit plant viruses [3,4,5]. Huge economic loss due to whitefly is a common phenomenon observed globally, including in India. The virus transmission ability and wide host adaptability of this pest make its management more difficult [6]. This has, in turn, led to heavy dependence on chemical pesticides for managing this notorious pest. Several agricultural practices, such as poor insecticide selection, substandard application techniques, and overdose of insecticide application against *B. tabaci*, have resulted in control failures in fields, along with the development of resistance to various organophosphates, synthetic pyrethroids, and neonicotinoid insecticides [7,8,9,10]. During the past several years, a high level of resistance in whitefly against a wide range of insecticides, including compounds of novel chemistry, has been reported from China [11], India [12] Iran [13], Israel [14], Italy [15], Malaysia [16], Pakistan [17], and the USA [18]. Nonetheless, persistent use of these toxic compounds for managing sucking pests, especially *B. tabaci*, has adverse effects on the environment and human health.

The molecular mechanisms underlying insecticide resistance reveal two basic principles, i.e., target site insensitivity and metabolic detoxification [19]. Primarily, metabolic detoxification may occur due to gene amplification, overexpression, or modification of the gene-coding proteins of major detoxifying enzymes, namely cytochrome P-450 s (P-450 s), glutathione S-transferases (GSTs), and others [20]. The P450s encoded by *cyp* genes, constituting a superfamily of enzymes [21,22], are known for their contribution to several imperative roles such as the growth, development, and detoxification of both endogenous and xenobiotic compounds [23]. Reports indicate the presence of around 600 P450s in insects belonging to 17 *cyp* families, and those in families 4, 6, 9, and 12 are associated with detoxification processes rendering tolerance to insecticides [22,24]. Furthermore, among the different cytochrome P450 genes, the *CYP6CM1* and *CYP6CX1* genes were found to be highly correlated with neonicotinoid resistance in a field population of whitefly in China [25]. Involvement of different cytochrome P450 genes in insecticide resistance has also been recorded in other insects, including *Musca domestica* and *Drosophila melanogaster*, against imidacloprid and DDT, respectively [26,27]. As insecticide resistance lowers the proficiency of chemical substances on the target pest [28], delineating the underlying mechanism of resistance in whitefly against different groups of insecticides, it has opened gateways for extensive research. However, the gene-mediated response alone cannot be held accountable for resistance in whitefly, as symbiotic bacteria may also have an important role in the detoxification of xenobiotic compounds.

Substantial evidence exists regarding microbe-mediated effects in whitefly, which confers several physiological benefits, including increased heat tolerance [29] and nutritional fitness [30] to mention a couple; however, studies exploring the relationship between symbionts and insecticide resistance are limited despite the link being indicated several decades ago [31]. For example, earlier reports suggest the presence of *Rickettsia* is instrumental in increasing the susceptibility of whitefly to insecticides such as thiamethoxam and acetamiprid [32]. Hence, it can be said that there exists a connotation between whitefly microbiota and insecticide resistance. All whitefly species harbor a diverse bacterial community. These comprise primary symbionts (P-symbionts) such as *Portiera*, which occur in all individuals located in certain specialized cells called bacteriocytes, and a multitude of secondary symbionts (S-symbionts) such as *Hamiltonella*, *Arsenophonus*, *Cardinium*, *Wolbachia*, and *Fritschea* present in hemolymphs, the midgut, etc. [33]. Unlike primary symbionts, which have a direct mutualistic relationship with the host, these secondary endosymbionts form a less stable partnership with their host and provide adaptive benefits to their partner, with effects being positive, negative, or neutral [34,35]. The involvement of these symbionts in protecting their host partners against thermal stress and many natural enemies is well documented [35,36], but the intricate interactions between symbionts and insecticide resistance have been less explored. Nonetheless, the exact mechanism underlying the associations between endosymbionts and insecticide resistance is still a matter of curiosity.

Keeping these points in mind, the current study was designed to evaluate the lethal effects of two neonicotinoids, imidacloprid and thiamethoxam, against adult whitefly populations. Changes in the bacterial microbiome community and expression pattern of two cytochrome P450 genes in imidacloprid- and thiamethoxam-treated whitefly populations were also monitored in parallel. Primarily, the following questions were addressed: (1) Do facultative and obligate symbionts respond differently to chemical stress imposed on whitefly? (2) How does the response of these symbionts vary with the time interval? (3) What are the changes in the expression pattern of different cytochrome P450 genes (*CYP6CM1* and *CYP6CX1*) in imidacloprid- and thiamethoxam-treated whitefly populations? Precisely, we discussed certain key research priorities, shedding light on the complex interaction between insect functioning, their microbial community, the insecticide-resistant gene, and their ability to tolerate xenobiotic compounds. An increasing number of studies indicate such complex interactions in a vast range of insects worldwide; however, this research experiment from India can be considered an important step in accentuating the possible mechanisms for the development of resistance in whitefly against prevailing insecticides used in the country, further suggesting novel management strategies of this pest.

## 2. Materials and Methods

### 2.1. Whitefly Rearing

The *B. tabaci* populations used in this study were collected from a research farm (C block) in BCKV, and a running culture of *B. tabaci* was maintained on eggplant seedlings (Samrat) under controlled environmental conditions at 27 ± 1 °C with 60% RH and 16 h light/8 h dark conditions.

### 2.2. Genetic Identification of Whitefly and Their Symbiont

The MtCoI gene was used for confirmation of whitefly with forward primer C1-J-2195 (5′-TTGATTTTTTGGTCATCCAGAAGT-3′) and reverse primer L2-N-3014 (5′ TCCAATGCACTAATCTGCCATATTA-3′) [37]. Total DNA was extracted using a Genomic DNA Isolation Kit (Sigma-Aldrich, St louis, USA) from 20 whitefly samples. The presence of four endosymbionts (*C. Portiera, Wolbachia*, *Arsenophonus*, and *Rickettsia*) was detected in the reared whitefly populations using their specific primers (Table 1) [38].

A PCR program was carried out in a total volume of 25 µL, containing 2 µL of template DNA, 12.5 µL of PCR master mix, 8.5 µL of molecular-grade water, and 1 µL each of forward and reverse primer specific to the symbiont. The thermal cycler programmed a denaturation step at 94 °C for 5 min, followed by 40 cycles at 94 °C for 30 s, annealing at different temperatures specific to the endosymbiont (60 °C for *Portiera*, 54 °C for *Arsenophonus*, 56 °C for *Wolbachia* and *Rickettsia*) for 30 s. Extension was carried out at 72 °C for 40 s with a final extension at 72 °C for 5 min.

### 2.3. Insecticides

Susceptibility of whitefly to two insecticides, namely imidacloprid 17.80 SL (Bayer, Pune, India) and thiamethoxam 25 WG (Syngenta, Thane, India), belonging to a neonicotinoid chemical group, was tested in the laboratory.

### 2.4. Bioassays

The toxicity of imidacloprid and thiamethoxam was evaluated against *B. tabaci* adults (5 days old) by following a modified leaf dip bioassay method of the Insecticide Resistance Action Committee [10]. To evaluate the toxicity, final doses were decided on a pre-experimental basis, and five dilutions, each of imidacloprid (3, 15, 30, 60, and 300 mg/L (ppm)) and thiamethoxam (1, 5, 10, 50, and 100 mg/L (ppm)), were prepared in tap water by the serial dilution method. In each treatment, eggplant leaves with petioles were dipped in the respective insecticide dilutions for 25 ± 2 s in a corning glass Petri plate (diameter = 15 cm) and air dried. The control leaves were dipped in water alone. After drying the solution on the surface of the leaves, the treated leaves were transferred to the corning glass Petri plate (diameter = 9 cm) containing 2% agar slants. Each treatment was replicated three times. In each replication, ten *B. tabaci* adults (30 in a treatment) were released, and plates were covered with ventilated lids. The experiment was conducted under laboratory conditions (temperature, 25 ± 2 °C; relative humidity, 75 ± 10%; and photophase, 14 h). The observations of the mortality were taken at 24, 48, 72, and 96 h after exposure (HAE). Moribund adults were considered dead.

### 2.5. Insecticide Treatment on Whiteflies

The whitefly population used in the experiment were fed leaves treated with sublethal concentrations, i.e., an LC_30_ concentration of imidacloprid and thiamethoxam at 24 h after exposure (HAE) taken from a previous bioassay. Whiteflies fed on untreated leaves were considered as control. The samples (surviving adults) collected at 24, 48, and 72 h (1, 2, and 3 days after exposure, i.e., 1 DAE, 2 DAE, and 3 DAE) during feeding on treated leaves were killed with the help of chloroform and stored at −80 °C for further analysis. The samples were subsequently used for extraction of DNA and RNA to determine the effect of chemical (insecticide) stress on the changes in the relative amount of symbionts, as well as on the expression pattern of two cytochrome P450 genes (*CYP6CM1* and *CYP6CX1*) using quantitative PCR (qPCR).

### 2.6. DNA Extraction

DNA was extracted from the adult whiteflies treated with imidacloprid and thiamethoxam at intervals of 1 DAE, 2 DAE, and 3 DAE separately by using a Genomic DNA Isolation Kit (Sigma-Aldrich, St Louis, USA). The purified DNA template was eluted in 40 μL of nuclease-free water supplied with the kit. Final products were assessed with the help of a BLBIO spectrophotometer (ELICO), and the eluted product was stored at −80 °C until use.

### 2.7. RNA Isolation

The total RNA was extracted from the imidacloprid- and thiamethoxam-treated whiteflies using an Insect RNA Isolation Kit (Thermo Fisher Scientific), following the manufacturer’s protocol. For each treatment, the RNA template consisted of 20 individual whiteflies that were eluted in 30 µL of molecular-grade water. RNA quality was evaluated using the Invitrogen^TM^ Qubit^TM^ 4 Fluorometer (Thermo Fisher Scientific, Waltham, USA) to determine the quality and quantity with high precision per microliter of RNA, and eluted templates were stored at −80 °C until use.

### 2.8. cDNA Synthesis

Synthesis of complementary DNA was performed by using the GeneSure H-Minus First-Strand cDNA Synthesis Kit (Genetix Biotech Asia Pvt. Ltd., New Delhi, India) by mixing 2.5 μL of total RNA with 1 μL of oligo dT, 1 μL of 10 mMdNTPs, and DEPC-treated water to a volume of 12 μL. The solution was incubated at 65 °C for 5 min, and the following reagents were added; 4 μL of 5X first-strand buffer, 1 μL of ribonuclease inhibitor (40 units/μL), and 4 μL of DEPC-treated water. This mixture was placed at 25 °C for 5 min before adding 1 μL of M-MLV RT. A final incubation at 42 °C for 60 min, followed by 70 °C for 15 min, was performed to terminate the reaction.

### 2.9. Quantitative PCR (qPCR) and Quantitative RT-PCR (qRT-PCR) Analysis

The expression of insecticide-resistant genes and the relative abundance of endosymbionts (*Portiera*, *Rickettsia*, *Arsenophonus*, and *Wolbachia*) were determined after exposure to xenobiotic stress at different time intervals using qPCR and qRT-PCR protocols, respectively. Then, 2X SYBR Green qPCR Master Mix (Applied Biosystems, Foster City, USA) was used. Primer names, their sequences, and annealing temperatures are mentioned in Table 1. The DNA samples were run in triplicate to ensure the validity of the data using the Agilent Technologies Stratagene Mx3000P Sequence Detection System. Amplification was carried out in a 10 µL reaction containing 5 µL of 2X SYBR Green PCR Master Mix, 0.5 µL of each primer (10 µM each), 1 µL of template DNA, 0.2 µL of ROX, and 2.8 µL of molecular-grade water. The following PCR cycling conditions were used: 3 min activation at 95 °C, followed by 40 cycles of 40 s at 95 °C, 40 s at 60 °C, and 45 s at 72 °C. All primers produced a single melt peak. An insect actin gene used as an internal control for normalization was run in parallel. The relative expression of each target was calculated by the 2^–ΔΔCtn^ method [39].

### 2.10. Statistical Analysis

To determine the LC values, the observations recorded on mortality were corrected using Abbott’s formula [40]. The data were subjected to probit analysis [41] using Microsoft Excel spreadsheets. The differences in the relative expression of insecticide-resistant genes and the relative amount of symbionts at different durations after insecticidal spray were analyzed using a one-way analysis of variance (ANOVA). The statistical significance of the difference between the means were compared by Tukey’s test at *p* < 0.05 and performed using SPSS 14.0 (SPSS Inc., Chicago, USA). The error bars present in the graphs represent standard error.

## 3. Results

### 3.1. Concentration Mortality Response of Imidacloprid and Thiamethoxam against Whiteflies

**Figure 1:**
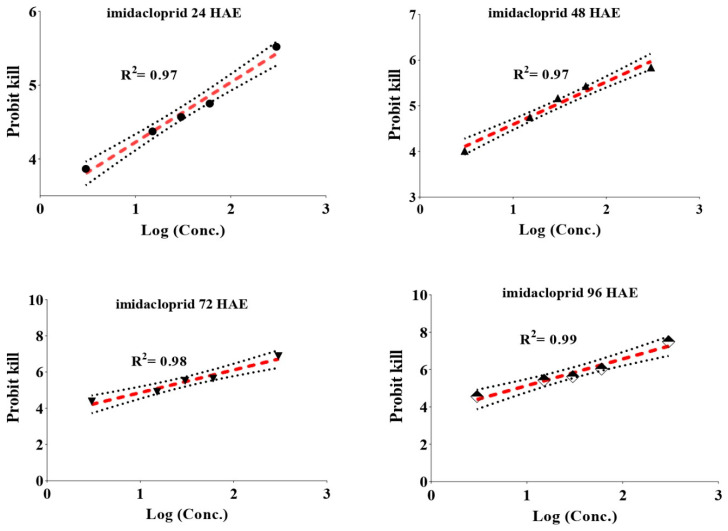
Concentration mortality response of imidacloprid against whitefly adults. The straight line represents the best fitting probit regression line between insecticide log dose and mortality probits (working probits) for adult *B. tabaci* at different intervals of time after the insecticide exposure, slopes of the lines are given in the Table 2. The arched line represents the standard error (SE) for the slope of straight regression line.

**Figure 2:**
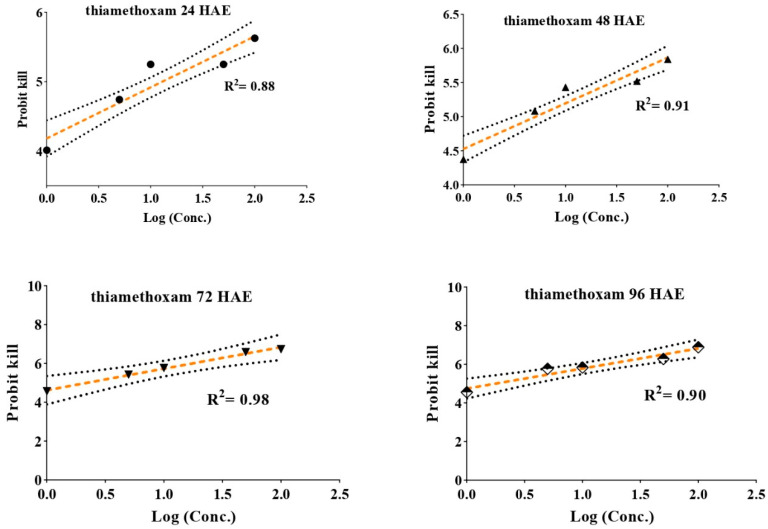
Concentration mortality response of thiamethoxam against whitefly adults. The straight line represents the best fitting probit regression line between insecticide log dose and mortality probits (working probits) for adult *B. tabaci* at different intervals of time after the insecticide exposure, slopes of the lines are given in the Table 2. The arched line represents the standard error (SE) for the slope of straight regression line.

Upon feeding of whitefly adults on insecticide-treated leaves for several hours, it was recorded that the adults were more susceptible to thiamethoxam than imidacloprid (Table 2). The median lethal dose showed that thiamethoxam (LC_50_ = 12.28 mg/L) was 7.29 times more toxic than imidacloprid (89.64 mg/L) at 24 h after exposure (HAE). Similarly, on increasing the duration of exposure, the toxicity at the median lethal dose for thiamethoxam was higher than imidacloprid; thiamethoxam (LC_50_ = 5.28, 2.34, and 1.73 mg/L) was 4.95, 5.44, and 4.15 times more toxic than imidacloprid (LC_50_ = 26.16, 12.73, and 7.18 mg/L) at 48, 72, and 96 HAE, respectively. The toxicity at the LC_30_ and LC_90_ levels also showed a similar trend. Slopes (b) at various hours varied from 0.813 to 1.094 for imidacloprid and from 0.708 to 0.961 for thiamethoxam at various intervals of exposure (Figure 1 and Figure 2).

### 3.2. Relative Change in Primary Endosymbiont (Portiera) after Application of Two Neonicotinoids

A comparative study involving changes in symbiont titers in whitefly populations after application of the two neonicotinoids measured at different time intervals revealed varying patterns in their quantitative analysis. The relative abundance of *Portiera* differed significantly after exposure to both the insecticides at different time intervals.

**Figure 3:**
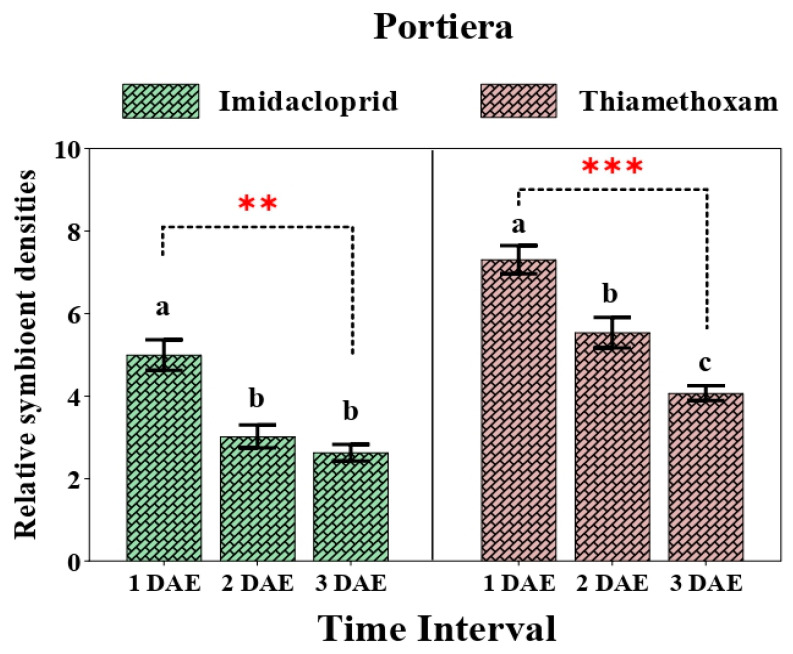
Relative density of *C. portiera* in whiteflies after exposure to sublethal doses of imidacloprid 17.80 SL and thiamethoxam 25 WG at various durations (1 DAE, 2 DAE, and 3 DAE). Bars represent the standard error of the mean (SEM), and the different letters indicate statistically significant differences between the treatments. *p* ≤ 0.01 is indicated by **, and *p* ≤ 0.001 is indicated by ***. DAE, days after exposure.

When whitefly adults were exposed to imidacloprid, the relative quantity of *Portiera* reduced (F_2,6_ = 21.30, *p* = 0.002) significantly after 2 days (4.9- to 3.02-fold), followed by a further decline in quantity (2.62-fold) after 3 days when compared with that of the control. However, changes in the relative amount of *Portiera* after 2 days and 3 days did not attain any statistical significance. By contrast, the relative amount of *Portiera* was always higher in the thiamethoxam-treated population compared to the imidacloprid-treated population. The relative titer of *Portiera* after 1 day was recorded to be higher (7.31-fold) and reduced significantly (F_2,6_ = 26.796, *p* = 0.001) after 2 days (5.53-fold) compared to that of the control and continued to decline as the duration increased (Figure 3).

### 3.3. Relative Change in Secondary Symbionts after Application of Two Neonicotinoids

The three secondary endosymbionts *Arsenophonus*, *Wolbachia*, and *Rickettsia* responded differently to insecticide treatment. In both imidacloprid- and thiamethoxam-treated whiteflies, the relative titer of *Arsenophonus* at different time intervals declined significantly (F_2,6_ = 71.335, *p* = 0.0001; F_2,6_ = 74.504, *p* = 0.0001).

**Figure 4:**
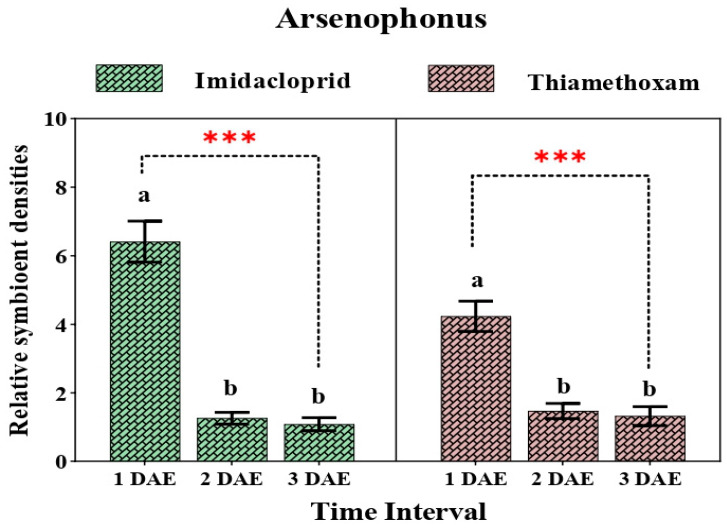
Relative density of *Arsenophonus* in whiteflies after exposure to sublethal doses of imidacloprid 17.8 SL and thiamethoxam 25 WG at various durations (1 DAE, 2 DAE, and 3 DAE). Bars represent the standard error of the mean (SEM), and the different letters indicate statistically significant differences between the treatments. *p* ≤ 0.001 is indicated by ***. DAE, days after exposure.

Upon exposure to imidacloprid, the relative quantity of *Arsenophonus* reduced dramatically after 2 days (6.41- to 1.25-fold), followed by a moderate decline in quantity (1.15-fold) after 3 days when compared with that of the control. Similarly, the relative abundance of *Arsenophonus* followed a similar pattern when the whitefly population was treated with thiamethoxam, i.e., an initial decline in quantity from 4.23- to 1.46-fold after 2 days with a further reduction after 3 days (1.43-fold) (Figure 4).

**Figure 5:**
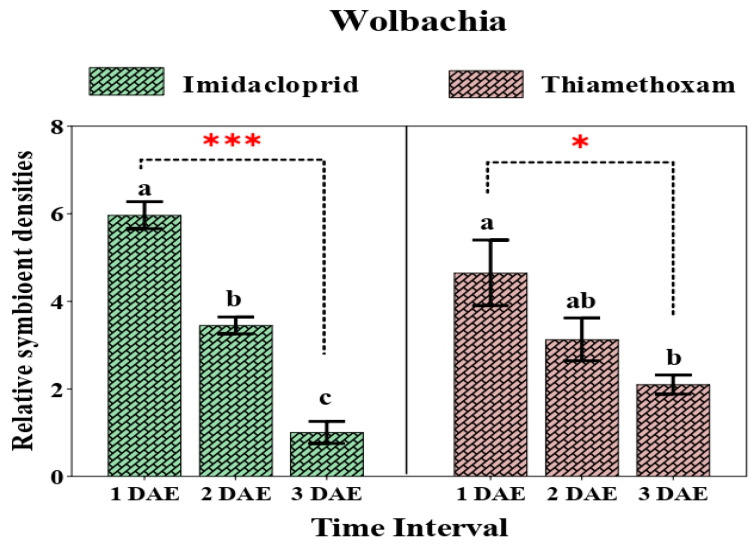
Relative density of *Wolbachia* in whiteflies after exposure to sublethal doses of imidacloprid 17.8 SL and thiamethoxam 25 WG at various durations (1 DAE, 2 DAE, and 3 DAE). Bars represent the standard error of the mean (SEM), and the different letters indicate statistically significant differences between the treatments. *p* ≤ 0.05 is indicated by * and *p* ≤ 0.001 is indicated by ***. DAE, days after exposure.

The relative abundance of *Wolbachia* varied significantly (F_2,6_ = 24.591, *p* = 0.001; F_2,6_ = 5.73, *p* = 0.041) in both the imidacloprid- and thiamethoxam-treated populations, respectively. After exposure to imidacloprid, the relative quantity of *Wolbachia* reduced (5.96- to 3.45-fold) after 2 days, followed by a dramatic decline in quantity (1.12-fold) after 3 days when compared with that of the control. Similarly, the relative abundance of *Wolbachia* followed a similar pattern over the course of time when the whitefly populations were treated with thiamethoxam, i.e., an initial decline in quantity from 4.65- to 3.13-fold after 2 days with a further reduction after 3 days (2.10-fold) (Figure 5).

**Figure 6:**
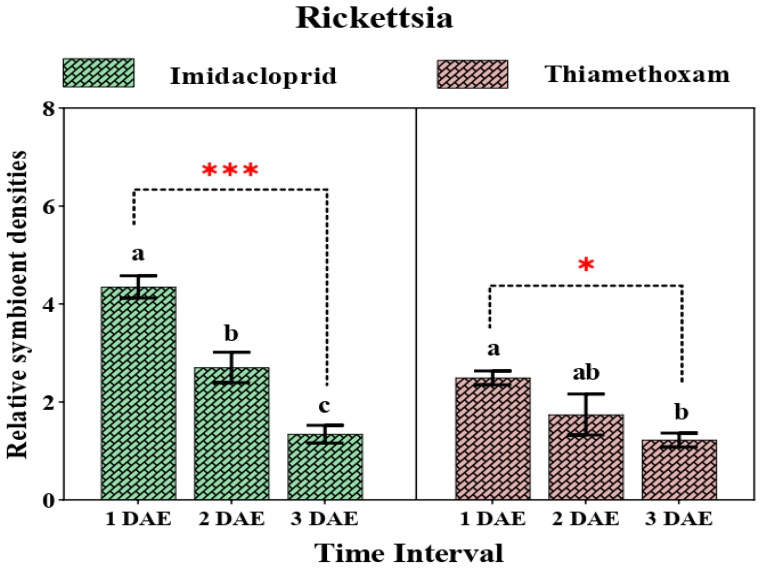
Relative density of *Rickettsia* in whiteflies after exposure to sublethal doses of imidacloprid 17.8 SL and thiamethoxam 25 WG at various durations (1 DAE, 2 DAE, and 3 DAE). Bars represent the standard error of the mean (SEM), and the different letters indicate statistically significant differences between the treatments. *p* ≤ 0.05 is indicated by * and *p* ≤ 0.001 is indicated by ***. DAE, days after exposure.

The relative amount of *Rickettsia* showed significant variation across different time intervals (F_2,6_ = 43.18, *p* = 0.0001; F_2,6_ = 5.53, *p* = 0.044) in both the imidacloprid- and thiamethoxam-treated populations, respectively. The relative titer of *Rickettsia* decreased over the course of different time intervals in the imidacloprid-treated population, i.e., 4.34- to 2.7-fold, after 2 days and continued decline up to 3 days of exposure. However, the reduction in the titer level of *Rickettsia* in the case of the thiamethoxam-treated population was relatively gradual, i.e., 2.4- to 1.74-fold, after 2 days, with a moderate reduction after 3 days of exposure (1.75-fold) (Figure 6).

### 3.4. Expression Pattern of Two Cytochrome P450 Genes after Exposure to Chemical Stress

**Figure 7:**
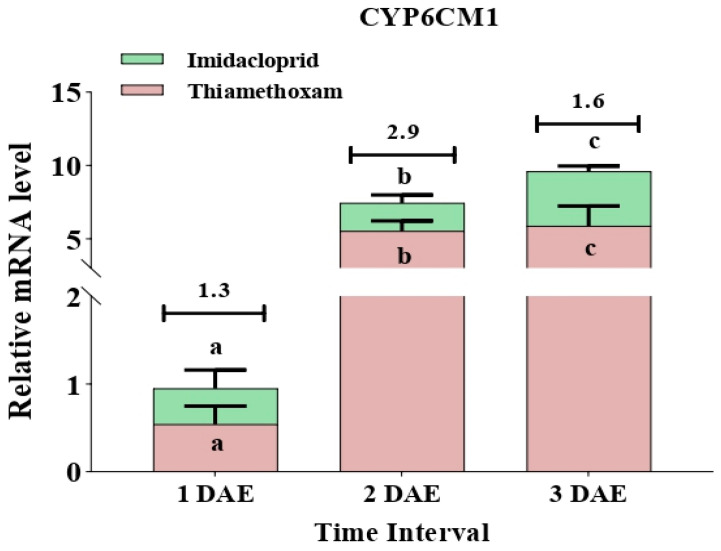
Expression of the insecticide-resistant gene (*CYP6CM1*) in whiteflies after exposure to sublethal doses of imidacloprid 17.8 SL and thiamethoxam 25 WG at various durations (1 DAE, 2 DAE, and 3 DAE). Bars represent the standard error of the mean (SEM), and the different letters above the symbols indicate a significant difference at the 0.05 level. DAE: days after exposure.

**Figure 8:**
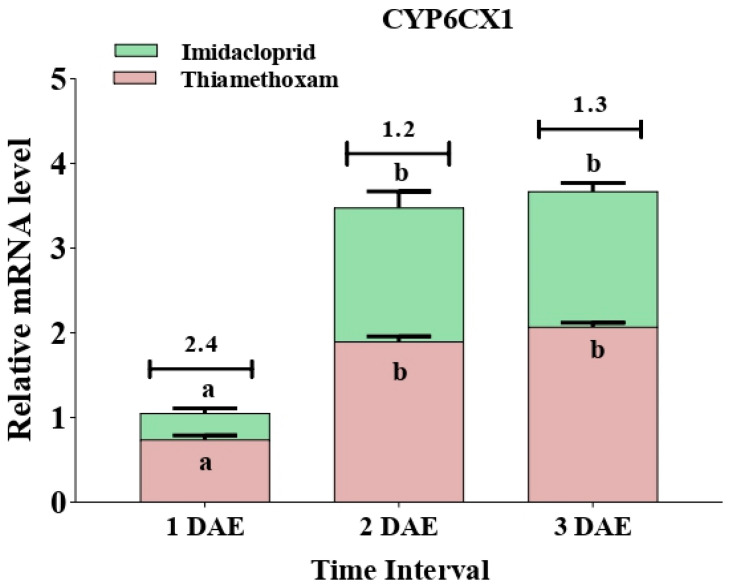
Expression of the insecticide-resistant gene (*CYP6CX1*) in whiteflies after exposure to sublethal doses of imidacloprid 17.8 SL and thiamethoxam 25 WG at various durations (1 DAE, 2 DAE, and 3 DAE). Bars represent the standard error of the mean (SEM), and the different letters above the symbols indicate a significant difference at 0.05 level. DAE: days after exposure.

The two cytochrome P450 genes, *CYP6CM1* and *CYP6CX1*, showed varying expression patterns when whitefly adults were subjected to imidacloprid and thiamethoxam treatment. The ratios of imidacloprid- to thiamethoxam-treated whiteflies in terms of transcript levels of CYP6CM1 were 1.3 at 1 DAE, 2.9 at 2 DAE, and 1.6 at 3 DAE (F_2,6_ = 11.06, *p* = 0.010; F_2,6_ = 16.95, *p* = 0.003). Similarly, insecticide treatment resulted in a higher expression of the *CYP6CX1* gene in the imidacloprid-treated population in comparison to thiamethoxam treatment. The ratios of imidacloprid- to thiamethoxam-treated whiteflies in terms of transcript levels of *CYP6CX1* were noted to be 2.4 at 1 DAE, 1.2 at 2 DAE, and 1.3 at 3 DAE (F_2,6_ = 144.66, *p* = 0.0001; F_2,6_ = 31.69, *p* = 0.001) (Figure 7 and Figure 8).

## 4. Discussion

Insects have evolved through lineages and established themselves successfully as eukaryotic life forms on planet Earth. They are classified depending on their nature, as some insects play a beneficial role by pollinating crops, while others have a detrimental effect on them and are a threat to food security worldwide [42]. Managing these insect pests is a massive task, and over the years, people have relied on the use of toxic molecules for checking pest incidence. As a result, insecticide resistance is a common phenomenon that has resulted in serious pest outbreaks and, consequently, even more losses [43,44]. Hence, insecticide resistance and subsequent management strategies are garnering much attention worldwide. As already mentioned, whitefly, known for causing serious damage to field and horticulture crops, is a difficult pest to manage that has also developed resistance against nearly 40 active ingredients of toxic compounds [45]. A shift from organophosphates and organochlorines to pyrethroids to neonicotinoids and other novel compounds has been ongoing over several years [46]. However, the failure of such insecticides against *B. tabaci* in India continues [8]. Naveen et al. [10] reported the insecticide resistance level of neonicotinoids for various genetic groups of whitefly in the Indian subcontinent by evaluating the median lethal doses of imidacloprid and thiamethoxam 96 HAE. The LC_50_ values for the various populations in the Indian subcontinent were reported to vary from 52 to 956 mg/L for imidacloprid and 26 to 194 mg/L for thiamethoxam, indicating the higher susceptibility of various population groups of *B. tabaci* to thiamethoxam compared to imidacloprid. Similarly, in the present study, the population was found to be more susceptible to thiamethoxam. The toxicity of imidacloprid was reported to be high compared to thiamethoxam against sucking pests, especially whitefly [47]. The factors, which demand more consideration in this regard, are insecticide-resistant gene expression, symbiont-mediated detoxification, or the role of gut symbionts, which might be accountable for differences in response against chemical stress [48].

We are aware of the different host–microbe interactions and their role in meeting their partners’ nutritional requirements and helping in ecological adaptation [49]. In recent times, there is growing awareness regarding the role of microbes in protecting their host partners from insecticides or different chemical compounds [50]. On exposure to insecticide stress, symbionts exert substantial effects on their hosts, which vary depending on the species of the host and symbiont, as well as the class or type of the toxic molecule [51,52,53,54,55]. Studies reveal infection with symbionts increased susceptibility of the hosts to insecticides [56,57,58]. To shed light on this facet, we elucidated the change in the relative amount of four endosymbionts in a lab population of whitefly upon the subjection of two neonicotinoid compounds at different time intervals. Recent findings reveal the involvement of primary symbionts in the defensive function of stinkbugs and psyllids [59,60]. On the other hand, the association of secondary symbionts with insecticide resistance has already been mentioned [56,61]. However, in the case of whitefly, the relationship between symbionts and insecticide resistance has been inconsistent.

Our findings reveal varying changes in the relative amount of primary and secondary symbionts upon spray of two different neonicotinoid compounds. In particular, the effect of two neonicotinoids on the titer level of the P-symbiont *Portiera* varied with the time interval. The relative abundance of *Portiera* was comparatively higher in the thiamethoxam-treated population in contrast with the imidacloprid-treated population, indicating that a higher amount of *Portiera* might be responsible for increased susceptibility of whitefly towards thiamethoxam. Another notable fact is that with increased duration of exposure to insecticides, the relative amount of *Portiera* gradually declined, suggesting that the increased uptake of toxic molecules by whiteflies might have deleterious effects on *Portiera*. On the contrary, the amount of *Rickettsia* was higher in the imidacloprid-treated population in comparison to the thiamethoxam-treated population. Our results showed good congruence with the findings of Pan et al. [62], who reported a higher amount of *Portiera* and a lower amount of *Rickettsia* in thiamethoxam-susceptible populations of whitefly. Nonetheless, previous studies also mention the association of *Rickettsia* with increased susceptibility of whitefly towards thiamethoxam and other such compounds [32,56]. Such incongruities are confusing but raise some vital questions that call for further attention. A possible explanation for such acrimonious results could be the different interactions between the symbiotic populations or the differences in host plants used in the studies.

On the contrary, the effect of imidacloprid and thiamethoxam on the relative amount of both *Wolbachia* and *Arsenophonus* was quite drastic within 1 day after exposure to both the toxicants. A possible reason behind such a steep fall might be their localization pattern. *Arsenophonus* is reported to be located in the salivary glands and midgut of *B. tabaci*, whereas *Wolbachia* displays a dual-localization pattern both inside and outside the bacteriosome [63,64], which might result in increased interaction between the xenobiotic compounds and these symbionts. The titer levels of *Arsenophonus* and *Wolbachia* were higher in the imidacloprid-treated whiteflies in comparison to those treated with thiamethoxam within 1 day after exposure; however, after 3 days, the thiamethoxam-treated populations harbored a higher amount of the symbionts, further suggesting that the relationship between symbionts and insecticides is highly variable. Not many studies in the literature mention the association of symbionts such as *Arsenophonus* and *Wolbachia* with insecticide resistance. However, few reports suggest the indirect involvement of *Wolbachia* in developing the susceptibility of insects to toxic compounds, as its density increases the physiological cost of resistance [65]. A clear understanding of the intricate relationship between symbionts and insecticide resistance is instrumental for the development of resistance management strategies.

Earlier studies have demonstrated that increased cytochrome P450 gene expression resulting in the enhancement of metabolic detoxification leads to resistance development against neonicotinoid compounds in whitefly [24]. Yang et al. found a positive correlation between expression of the *CYP6CM1* gene and imidacloprid resistance in field populations of whitefly in China [25]. Our studies also revealed a higher expression of the *CYP6CM1* and *CYP6CX1* genes in imidacloprid-treated whitefly compared to that of the thiamethoxam-treated population. A higher expression of insecticide-resistant genes might have resulted in increased resistance of whitefly towards imidacloprid. In other insects such as *Musca domestica*, overexpression of P450 genes such as *CYP6A1* and *CYP6D1* was associated with resistance to organophosphates, pyrethroids, DDT, and other juvenile hormone analogs [66]. Zhuang et al. also reported a positive correlation between the expression of *CYP6CX1* and imidacloprid resistance in a field population of whitefly [67]. Our results indicate a much stronger association between imidacloprid and the two P450 gene expressions in *B. tabaci*, but this also calls for functional studies of the protein encoded by the genes in order to ratify its role in detoxifying imidacloprid.

## 5. Conclusions

In summary, this paper provides a deeper insight into the role of symbionts in developing insecticide resistance. By synergizing our understanding of the insect–bacteria interactions with the urgent need to control pest populations, there is a possibility of developing symbiont-targeted pesticides in the near future. This could be a better substitute for the disposal of harmful antibiotics into the environment. We also gain an understanding of the two P450 genes and their direct positive correlation with imidacloprid resistance in whitefly populations. This understanding can be used for deploying RNA-silencing techniques to silence the target gene and increase the efficacy of the insecticides used.

## Figures and Tables

**Table 1:** List of primers used in the study.

**Organism**	**Accession Number**	**Primer Name**	**Primer Sequences (5′** **→** **3** **′** **)**	**Annealing Temperature (°C)**
**Diagnostic PCR**				
“*Ca. Portiera aleyrodidarum*”	MT585785	Por-F Por-R	CGTACGGAAACGTACGCTAA TAAGCATAGGGCTTTCACATAAA	60
*Rickettsia* sp.	MT027499	Ric-F Ric-R	GCTCAGAACGAACGCTGG GAAGGAAAGCATCTCTGC	56
*Wolbachia*	MT 032316	Wol-F Wol-R	CGGGGGAAAATTTATTGCT AGCTGTAATACAGAAAGGAAA	56
*Arsenophonus*	MT026928	Arse-F Arse_R	CGTTTGATGAATTCATAGTCAAA GGTCCTCCAGTTAGTGTTACCCAAC	54
*B. tabaci*	PRJEB41468	C1-J-2195 L2-N-3014	TTGATTTTTTGGTCATCCAGAAGT TCCAATGCACTAATCTGCCATATTA	53
**qPCR**	**Target Gene**	**Primer Name**	**Primer Sequences (5′** **→** **3** **′** **)**	**Annealing Temperature (°C)**
*“Ca. Portiera aleyrodidarum”*	16 S rDNA	Port73-F Port266-R	TAGTCCACGCTGTAAACG AGGCACCCTTCCATCT	60
*Rickettsia* sp.	gltA	glt375-F glt574-R	AAAGGTTGCTCATCATGCGTT GCCATAGGATGCGAAGAGCT	60
*Arsenophonus*	23 S rDNA	23 S-F 23 S-R	CGTTTGATGAATTCATAGTCAAA GGTCCTCCAGTTAGTGTTACCCAAC	60
*Wolbachia*	Wsp	Wsp-F Wsp-R	TGGTCCAATAAGTGATGAAGAAAC AAAAATTAAACGCTACTCCA	60
*B. tabaci*	CYTP6CM1	CM1-F CM1-R	CACTCTTTTGGATTTACTGC GTGAAGCTGCCTCTTTAATG	60
*B. tabaci*	CYTP6CX1	CX1-F CX1-R	GTGCCCTACATCTCGCCTATC CATTTCTTTCGTCGTCTCCAAC	60
*B. tabaci*	β-actin	Actin-F Actin-R	ACCGCAAGATTCCATACCC CGCTGCCTCCACCTCATT	60

**Table 2:** Toxicity of imidacloprid and thiamethoxam against whitefly.

Insecticide	HAE	LC Values (mg/L)			χ2	Slope ± SE	95% CL at LC_50_ (mg/L)
		LC_30_	LC_50_	LC_90_			
Imidacloprid	24	20.3	89.64	3379.55	0.528	0.813 ± 0.178	48.09–250.28
	48	7.11	26.16	630.61	0.843	0.927 ± 0.179	14.39–45.61
	72	4.19	12.73	192.04	0.801	1.087 ± 0.194	6.73–20.46
	96	2.382	7.18	106.46	0.215	1.094 ± 0.206	3.14–12.06
Thiamethoxam	24	2.23	12.28	794.76	3.012	0.708 ± 0.156	5.72–26.03
	48	0.872	5.28	430.35	1.239	0.671 ± 0.155	1.76–10.89
	72	0.631	2.34	57.53	0.622	0.921 ± 0.175	0.86–4.33
	96	0.492	1.73	37.21	2.616	0.961 ± 0.183	0.58–3.27

HAE = hours after exposure; df = 3, number of individuals treated = 30. The confidence limit (CL) for lethal concentration (LC) values was 95%.

## Data Availability

The sequence determined as part of this study has been deposited in GenBank under accession numbers MT585785, MT027499, MT032316, MT026928 and PRJEB41468.
